# Supplementary material for: Development of a Mortality Prediction Model in Hospitalised SARS-CoV-2 Positive Patients Based on Routine Kidney Biomarkers
Source: Int J Mol Sci. 2022 Jun 30;23(13):7260. doi: 10.3390/ijms23137260 (PMC9266863; doi:10.3390/ijms23137260)
Supplement: Supplementary file 1 [file ijms-23-07260-s001.zip › ijms-1720750-supplementary.pdf]

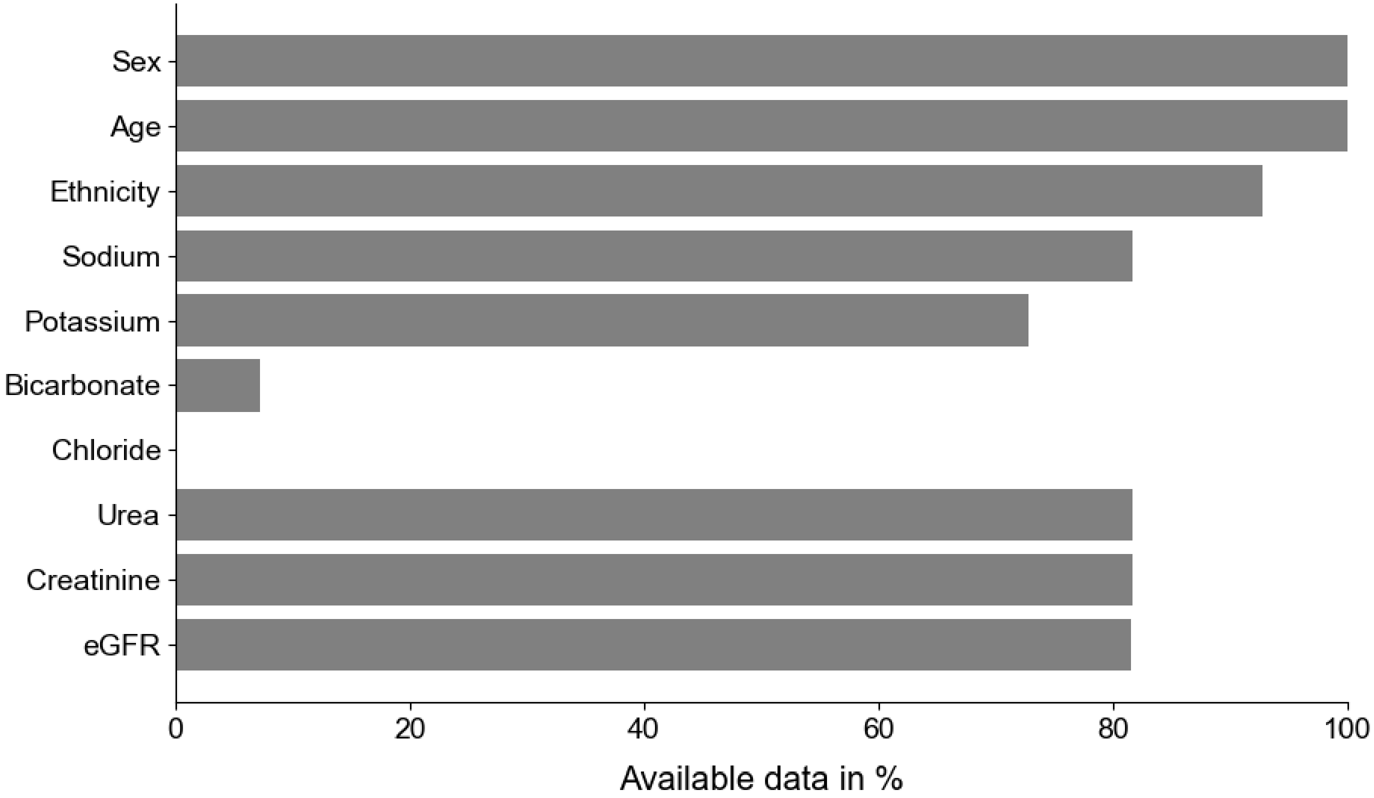

Figure S1. Density map of candidate predictors. Only predictors with at least 60% completeness were included in the model. Predictors with less than 40% available data were excluded. eGFR: estimated glomerular filtration rate.

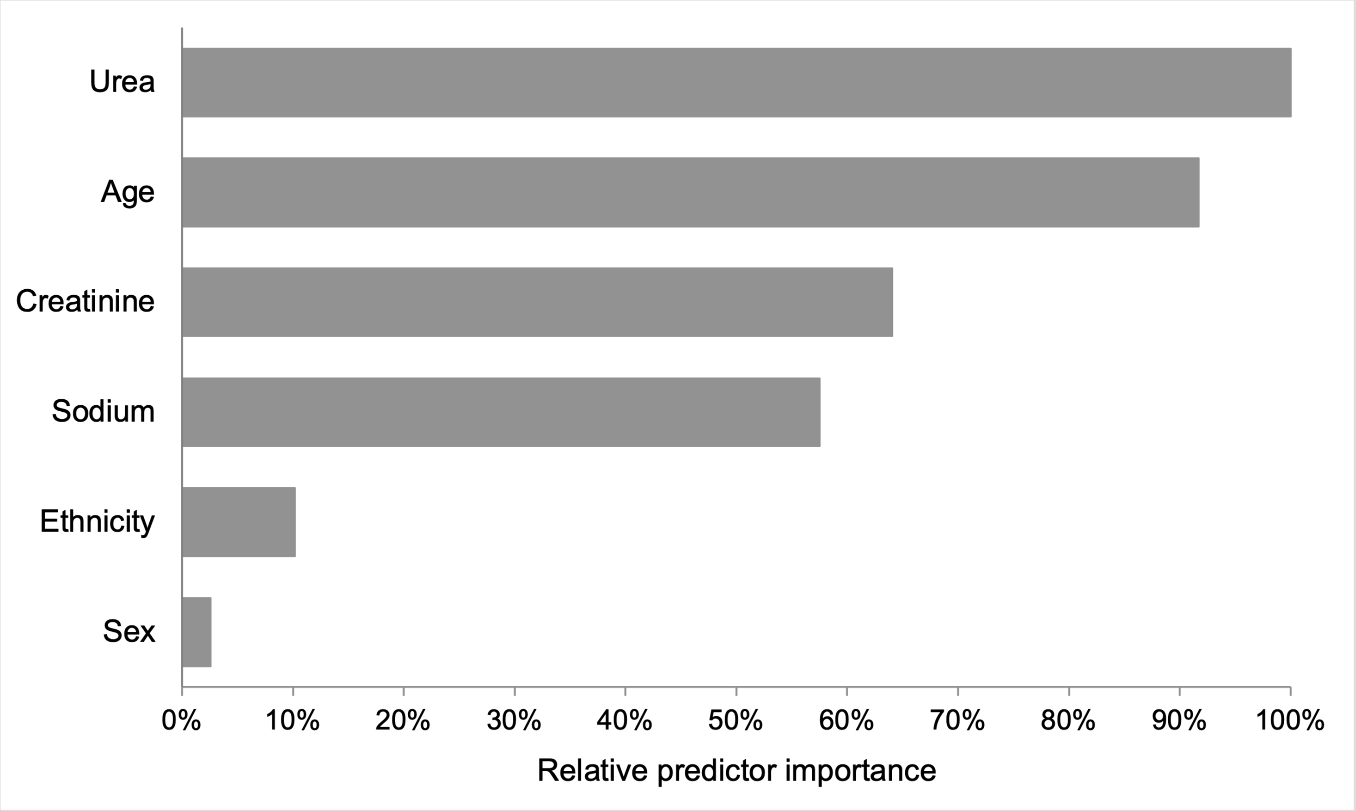

Figure S2. Relative importance of the predictors in the Random Forest model. The importance was relative to the most important predictor 'Urea'.

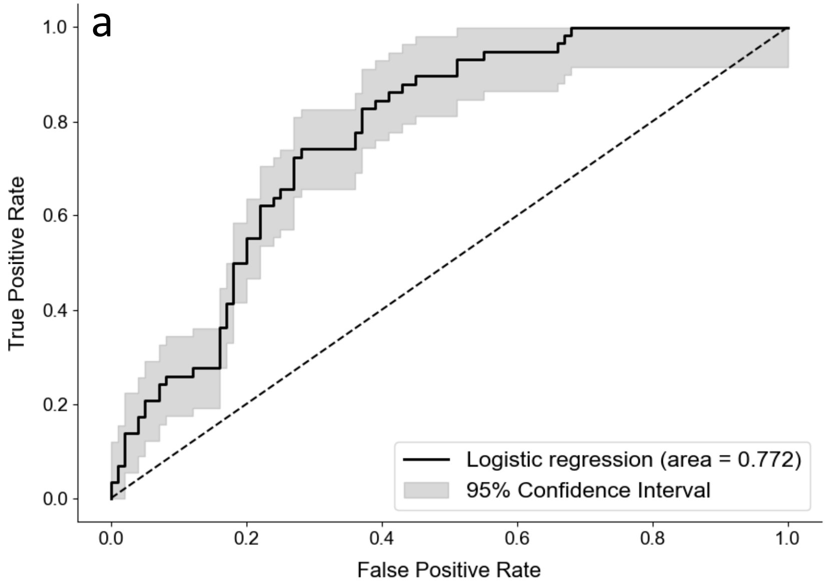

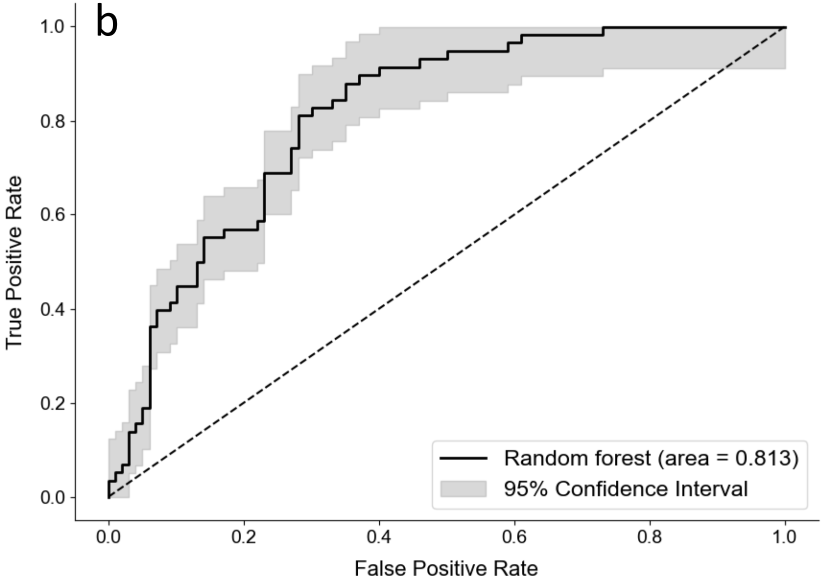

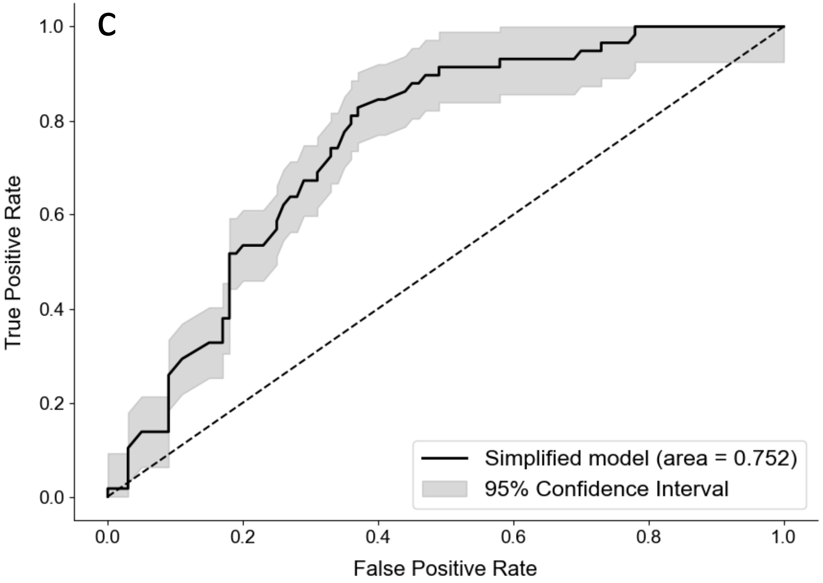

Figure S3. Area under receiver operating characteristic curve in the development cohort for (a) Logistic Regression, (b) Random Forest model, and (c) simplified model.

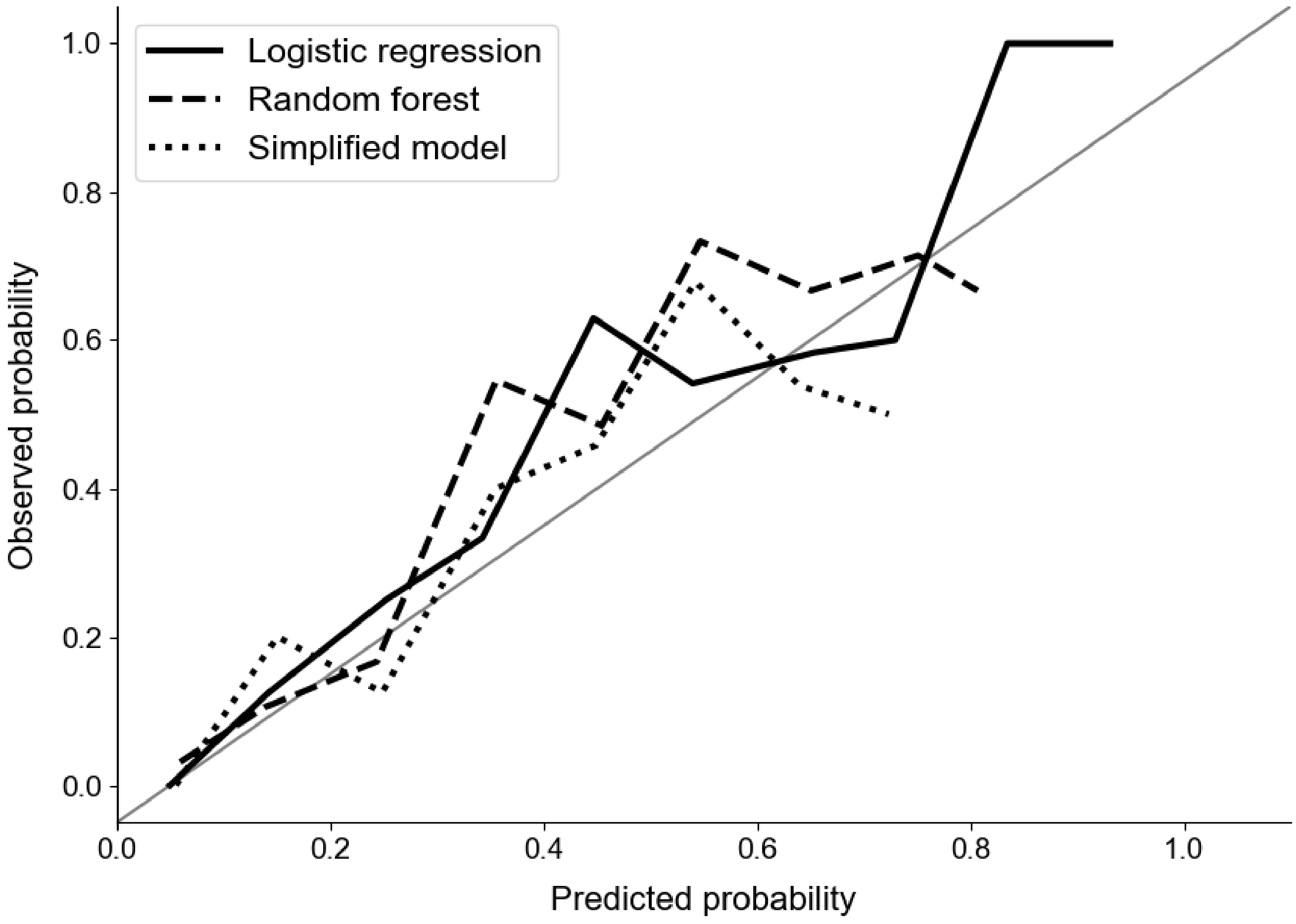

Figure S4. Calibration curves for the Logistic Regression, Random Forest, and simplified model in the development cohort.

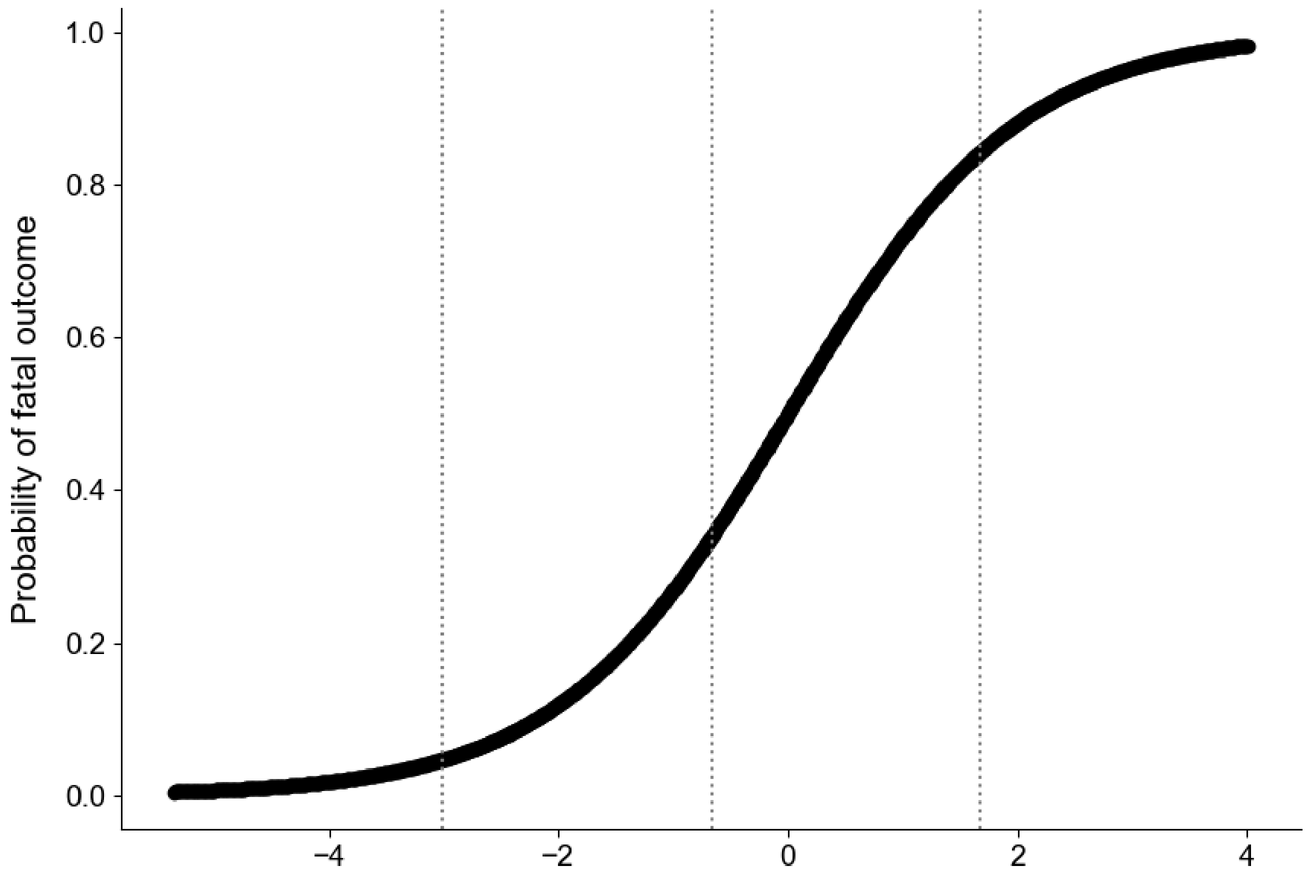

Figure S5. Sigmoid curve of logistic regression model with predictors sex, age, ethnicity, sodium, and urea. The grey dotted vertical lines show the cut-off values for the four risk groups.
